# Quantifying phenotype-environment matching in the protected Kerry spotted slug (Mollusca: Gastropoda) using digital photography: exposure to UV radiation determines cryptic colour morphs

**DOI:** 10.1186/s12983-017-0218-9

**Published:** 2017-07-10

**Authors:** Aidan O’Hanlon, Kristina Feeney, Peter Dockery, Michael J. Gormally

**Affiliations:** 1 Applied Ecology Unit, School of Natural Sciences, National University of Ireland Galway, Galway, Ireland; 2 Centre for Microscopy and Imaging, National University of Ireland Galway, Galway, Ireland

**Keywords:** Camouflage, Mollusc, Phenotypic plasticity, Pigmentation, UV radiation, Animal colouration, Digital photography, Disruptive patterning, Gastropoda, Polyphenism, Slug, Terrestrial mollusc, Visual predation

## Abstract

**Background:**

Animal colours and patterns commonly play a role in reducing detection by predators, social signalling or increasing survival in response to some other environmental pressure. Different colour morphs can evolve within populations exposed to different levels of predation or environmental stress and in some cases can arise within the lifetime of an individual as the result of phenotypic plasticity. Skin pigmentation is variable for many terrestrial slugs (Mollusca: Gastropoda), both between and within species. The Kerry spotted slug *Geomalacus maculosus* Allman, an EU protected species, exhibits two distinct phenotypes: brown individuals occur in forested habitats whereas black animals live in open habitats such as blanket bog. Both colour forms are spotted and each type strongly resembles the substrate of their habitat, suggesting that *G. maculosus* possesses camouflage.

**Results:**

Analysis of digital images of wild slugs demonstrated that each colour morph is strongly and positively correlated with the colour properties of the background in each habitat but not with the substrate of the alternative habitats, suggesting habitat-specific crypsis. Experiments were undertaken on laboratory-reared juvenile slugs to investigate whether ultraviolet (UV) radiation or diet could induce colour change. Exposure to UV radiation induced the black (bog) phenotype whereas slugs reared in darkness did not change colour. Diet had no effect on juvenile colouration. Examination of skin tissue from specimens exposed to either UV or dark treatments demonstrated that UV-exposed slugs had significantly higher concentrations of black pigment in their epithelium.

**Conclusions:**

These results suggest that colour dimorphism in *G. maculosus* is an example of phenotypic plasticity which is explained by differential exposure to UV radiation. Each resulting colour morph provides incidental camouflage against the different coloured substrate of each habitat. This, to our knowledge, is the first documented example of colour change in response to UV radiation in a terrestrial mollusc. Pigmentation appears to be correlated with a number of behavioural traits in *G. maculosus*, and we suggest that understanding melanisation in other terrestrial molluscs may be useful in the study of pestiferous and invasive species. The implications of colour change for *G. maculosus* conservation are also discussed.

**Electronic supplementary material:**

The online version of this article (doi:10.1186/s12983-017-0218-9) contains supplementary material, which is available to authorized users.

## Background

The ability of many animal species to accurately match surrounding habitat features (i.e. camouflage) has been held up as strong evidence of natural selection since Darwin’s time [1]. Intraspecific phenotypic variation is common within many animal populations and a consistent pattern of phenotype-environment matching and disruptive markings can be considered an indication that a particular phenotype is adaptive [2]. Such patterns of phenotype-environment matching in animal colouration are most often discussed in the context of predator avoidance and it has been shown that disruptive patterns, as well as background colour matching, can prove highly effective in concealing an animal [3]. The role of predation, therefore, in maintaining variation in cryptic prey colour morphs and patterns is relatively well-understood [4]. Apart from concealment from predators, however, animal colours and patterns may serve other functions such as thermoregulation or social signalling. Thus, not all apparently cryptic morphs are the sole result of directional selection by predators over many generations and, for some species, external cues can stimulate developmental or behavioural mechanisms which lead to rapid adaptation within the lifetime of an individual (i.e. phenotypic plasticity). Plasticity in animal crypsis has been demonstrated in response to a wide range of cues such as diet [5, 6], seasonal temperature changes [7], illumination and visual background properties [8, 9]; detection of a possible predation threat [10], and ultraviolet (UV) radiation [11, 12].

Colour variation appears to be maintained by frequency-dependent selection for many gastropod species (e.g. in land snails [13, 14]; in littorinid snails [15, 16]). Differences in gastropod colour morphs can also arise as adaptations to climatic selection where relatively darker or lighter shell and skin pigmentation evolves within populations exposed to cooler or warmer climates, respectively (e.g. as in littorinids [17, 18]; in Western Irish *Cepea nemoralis* L [19]; and in horn snails [20]). Skin colouration in terrestrial slugs can be highly variable, even within populations [21]. The mechanisms involved in determining slug colouration most likely vary with species and local adaptations may arise within populations of slugs exposed to different environmental conditions. Pigmentation has been explained as a simple Mendelian-inherited trait for some species of slug [22, 23]. Skin colouration and mottling (possibly functioning as crypsis) has been shown to be under polygenic control for *Limax flavus* L and *Limacus maculatus* Kaleniczenco [24]. Colour variation can also arise as a result of hybridization with closely related species (e.g. as in *Arion ater* L and *Arion rufus* L [25]). However, there is also evidence that different colour morphs in slug species can arise as local adaptations to different environmental conditions. While Evans [25], for example, found similar isozyme profiles between colour morphs of *Arion ater* agg., Taylor [21], in a robust survey of the malacofauna of Ireland and Britain, observed that dark colour morphs were associated with higher altitudes and cooler, wetter climates, suggesting climatic selection for pigmentation. Chevallier [26] provided further evidence of climatic selection for *A. ater* agg. and observed a similar pattern for *Arion lusitanicus* Mabille, with darker morphs prevailing at altitudes above 500 m. Jordaens et al. [27], on the other hand, demonstrated that diet can also influence skin pigmentation in F_1_ offspring of three species of arionid slug, resulting in a loss of ‘species-specific’ colour characteristics. The Kerry spotted slug *Geomalacus maculosus* (Allman) is unusual in that it appears to possess disruptive patterning as well as background-matching colouration which may provide different degrees of camouflage in alternative environments. This EU-protected species is associated with forested and open habitats (blanket bog and mountain heath) in Ireland where it occurs in one of two distinct colour morphs. In forested habitats *G. maculosus* generally possesses a hazel brown to ginger brown body colour with white and yellowish spots which appear to accurately match the moss and lichen-covered bark of trees. In open habitats, on the other hand, the slug generally possesses a dark blue-grey to black body colour with white spots where it seems to accurately match lichen-covered boulder outcrops which it uses for shelter and feeding [21, 28–31]. Little is known about population structure of *G. maculosus* and whether this affects colouration. Reich et al. [32] studied the population genetics of *G. maculosus* on which basis they suggested that the species originated in northern Spain 15Myr ago. No differences in 16S rRNA or COI genes were found between black and brown colour morphs (I. Reich, *pers*. *comm*.). Furthermore, newly hatched juveniles are brown in both forested and open habitats [31], suggesting that body colouration in this species may be a plastic trait.

This study was undertaken to test the hypothesis that each colour morph of *G. maculosus* is habitat-specific and provides camouflage. We used standardized digital photography to quantify widespread phenotype-environment matching in this internationally important mollusc species. Experiments were also conducted with laboratory-reared juvenile slugs to determine whether different environmental conditions (UV exposure or diet) could induce colour-change and whether this may help to elucidate the function of each colour morph observed in the wild. The study will also help inform the debate regarding potential translocation of the species as a possible mitigation measure within the Environmental Impact Assessment process.

## Methods

### Study sites and animal sampling

**Fig. 1:**
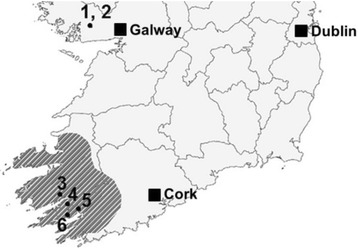
Partial map of Ireland showing survey sites (black circles) inside the distribution range of *G. maculosus* (shaded area). Conifer (1) and blanket bog (2) habitats at Oughterrard, Co. Galway; conifer habitat at Tooreenafersha, Co. Kerry (3); blanket bog habitat adjacent to Uragh woods, Co. Kerry (4); Oak forest habitat at Glengarriff Nature Reserve, Co. Cork (5) and blanket bog at Leahill Bog, Co. Cork (6). Black squares show the locations of Galway, Cork and Dublin cities for reference. Map modified from G. Kindermann, 2016©

Free-living slugs were photographed from six sites in Ireland across the western counties of Galway, Kerry and Cork (Fig. 1). Sites were selected on the basis that previous surveys had found high numbers of Kerry slugs in these areas [30, 33, 34, 35]. Forested sites were a combination of conifer plantations and oak woodlands (Fig. 1: sites 1, 3 and 5), and open habitats surveyed were blanket bog areas (Fig. 1: sites 2, 4 and 6). Refuge traps (De Sangosse, France) were used to collect slugs. These traps are 50 cm × 50 cm sheets of absorbent material covered with a reflective upper surface and a perforated dark lower surface which maintains a damp, cool environment beneath the trap. This method has been shown to be an effective technique for live-trapping *G. maculosus* [36]. Traps were placed on *Q. petraea* Liebl tree trunks (*n* = 4) at breast height (approx. 1.5 m) at site 5; on sandstone boulder outcrops at sites 4 (*n* = 4) and 6 (*n* = 4); and on granite outcrops at site 2 (site descriptions given in Additional file 1: Table S1). Each trap was checked for slugs one month after they had been set. Traps on sitka spruce *Picea sitchensis* Carr. trees at sites 1 and 3 were set previously by other researchers in the Applied Ecology Unit, NUI Galway, as part of separate projects [34, 35]. Each trap was only checked once for slugs to avoid pseudoreplication, except for site 1. Slugs in this site were removed from tree trunks after they had been photographed for use in a separate behavioural study, so multiple trips were possible since we could be positive that we were not taking pictures of the same individuals. Any additional slugs found near the traps were also photographed and included in colour analyses and subsequently removed from the site for use in a separate study. Slugs were collected with permission from the National Parks and Wildlife Service, Department of Arts, Heritage and the Gaeltacht (Licence No. C097/2015).

### Quantification of G. maculosus colour using digital photography

Digital photography was used to estimate the degree of phenotype-environment matching of adult slugs at each site, following a simplified version of the suggested methodology outlined in Stevens et al. [37]. A colour-checker card (X-Rite, Munsell Color Laboratories) was used to standardize the reflectance values obtained from digital photographs. The card consists of 24 coloured squares which are manufactured to represent common natural colours and a six-step greyscale from white to black. The ‘adjacent’ method validated by Bergman and Beenher [38] was used, whereby slugs were photographed in the same image as the colour-checker card. The camera used was a Nikon D3000 digital single-lens reflex camera with a pixel count of 10.2 megapixels and full control over metering and exposure. All photos of wild slugs in this study were taken at F-stop: f/5.6 with a shutter speed of 1/100 s, ISO 800. Images were saved to the camera memory card as uncompressed Nikon Electronic Format (NEF) raw image files. After transferring all files to a computer, Adobe Photoshop was used to convert the raw NEF files to 8-bit Tagged Image File Format (TIFF) files, for compatibility with GIMP 2.0 image processing software. White balance was corrected in GIMP 2.0 to standardize each photo with reference to the white square of the colour-checker card, such that this white square was equal to a reflectance score of 255 in R, G and B colour channels (i.e. ‘true’ white) for each image.

**Fig. 2:**
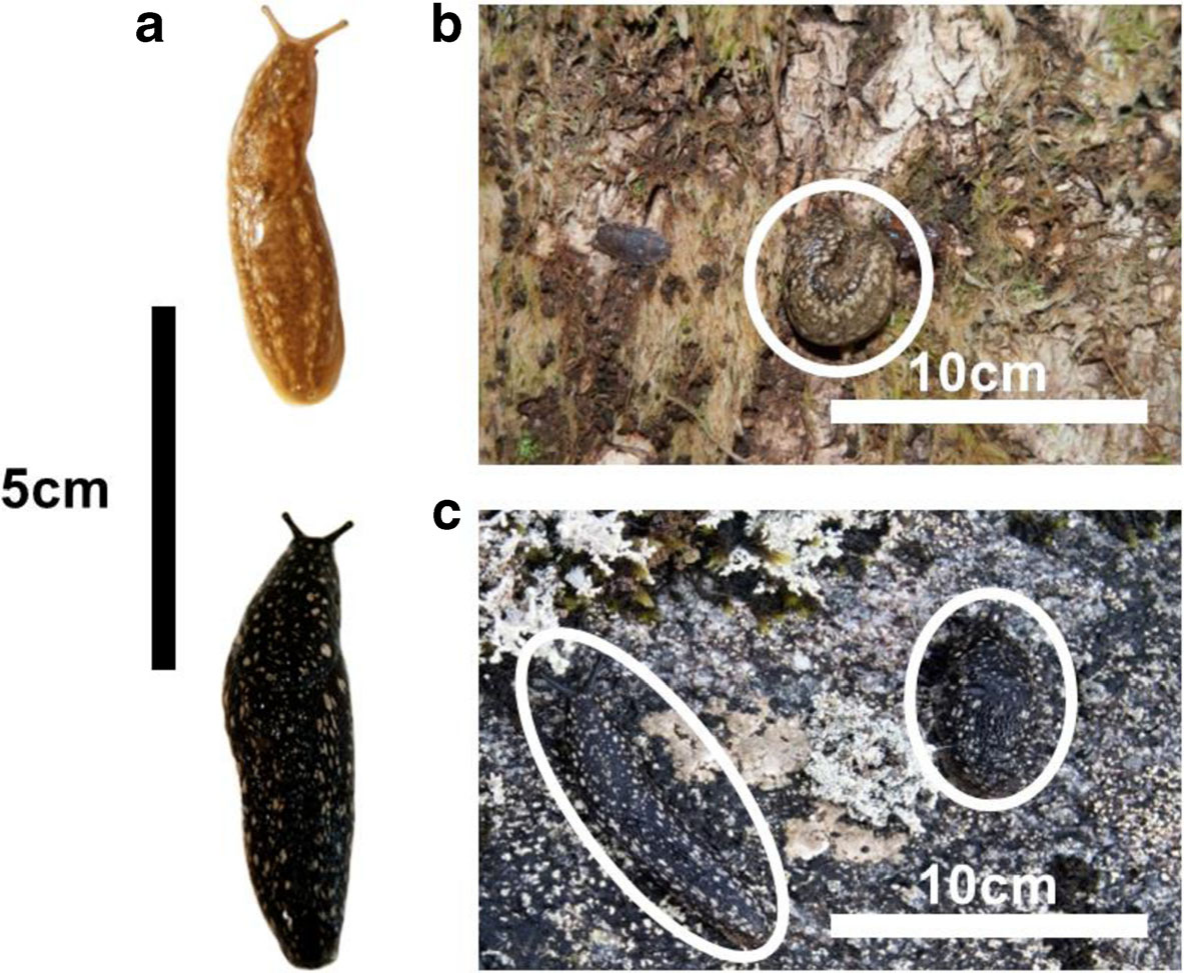
**a** Each colour morph of *G. maculosus* was photographed on its natural substrate on tree trunks in forested sites (**b**) or on boulder outcrops in blanket bog (**c**) alongside a colour standard card. Reflectance in R, G and B colour channels was calculated from a 1 cm × 1 cm square drawn over the mantle to estimate slug colouration, and a further three squares measuring 3 cm × 3 cm were drawn over patches of the substrate to calculate background R, G and B reflectance. Scale bars were measured from original photographs

To quantify colouration of individual slugs, a 1 cm × 1 cm square was drawn over the mantle of the slug in each image. Mean R, G and B reflectance values (calculated in-program by GIMP 2.0) were then recorded per square. To measure substrate colouration, three additional 3 cm × 3 cm squares were placed over background substrate in the photographs along-side each animal and the mean R, G and B reflectance scores of these three squares was calculated in-program. This was to determine whether *G. maculosus* phenotypes match a random sample of their background substrate (Fig. 2). The same method was used to quantify colour of juvenile slugs in UV-exposure and feeding experiments (outlined below). Due to the small body size of newly-hatched slugs, a 0.5 cm × 0.5 cm square was instead used to calculate mean R, G and B values over the mantle of juvenile slugs. Juvenile slugs were photographed once per month in the laboratory at F-stop f/4 with a shutter speed of 1/100 s, ISO 800.

### Effect of UV-exposure, darkness and diet on pigmentation

In addition to studying habitat-phenotype matching in wild-caught adult slugs, experiments were performed with newly hatched juveniles to investigate whether UV-exposure or diet can influence body colouration.

To investigate whether UV radiation could affect pigmentation in *G. maculosus*, hatchlings (*n* = 30) were randomly assigned to either a UV or ‘darkness’ treatment. For UV treatments, juveniles (*n* = 15) were kept in the laboratory in a clear plastic container (37 cm × 30 cm × 25 cm) fitted with a cold 13-watt fluorescent UVB bulb (Exo Terra, Canada). The bulb was fixed in the container lid at a distance of 25 cm from the container floor, resulting in a mean UVB irradiance value of approximately 25 μW/cm^2^ (estimated from information provided with purchase of the bulb) on the container floor. The floor of each container was lined with a sheet of wet cotton wool covered with tissue paper and each container lid was sealed with ParaFilm® to prevent the slugs from dehydrating. A small shelter constructed from laminated cardboard was placed on one side of the container and food (porridge oats) was placed on the opposite side. This was to ensure that the slugs had shelter from excessive UV radiation and that they would be forced to periodically leave the shelter to feed. The UV light was set on a 14:10 dark: light cycle which approximated with the natural photoperiod when experiments began in February 2016 in a laboratory which also received ambient light. For ‘darkness’ treatments, juvenile slugs (*n* = 15) were kept in identical conditions but the containers were not fitted with a UV light source and were kept in constant darkness. Juveniles for each treatment came from two egg-batches laid within the same week which were split in half with each hatchling assigned at random to one of two treatments. Both egg batches were laid in February 2016 by two captive adults of the brown phenotype.

In feeding trials, newly hatched juveniles (*n* = 45) were randomly assigned to one of three plastic containers (17 cm × 11 cm × 6 cm) where they were fed one of three food types: organic carrot (*n* = 15 slugs), spinach (*n* = 15 slugs) or porridge oats (*n* = 15 slugs). The container floor was lined with a layer of moist tissue paper to maintain damp conditions and protect the slugs from dehydration. Cotton wool was not necessary to maintain adequate moisture in feeding trial boxes (as in the UV and darkness trials) due to their smaller size. The tissue was re-misted three times per week and decaying food was replaced as necessary (typically once per week). The containers were housed in the laboratory on a shelf approximately 5 m from a SW-facing window providing identical natural photoperiod cues to each diet group. Juveniles used in feeding trials originated from egg batches laid in the laboratory in September 2015 by two captive adults of the brown phenotype.

### Stereological estimation of epithelial pigment

At the end of the UV / dark trials, all remaining slugs (*n* = 6 of each colour morph) were sacrificed using chloroform vapour and 1 cm × 1 cm skin samples from the slug mantle (effectively all the mantle) were removed following Rowson et al.[31]. Skin samples were fixed in 4% paraformaldahide before being dehydrated and embedded in paraffin wax. Sections (5 μm thick) were then cut on a Leica RM2125RT microtome and stained with hematoxylin/eosin. Transverse sections (1 per individual: 6 UV-exposed and 6 darkness-reared slugs) were imaged under a Leica DM500 microscope. Simple point-counting methods were used to estimate the volume fraction of pigment to the epithelium (mean of 16 grid samples per individual: mag. ×800). Mean epithelial thickness was also estimated (from 10 measuring points per individual: mag. ×200). Estimates of the volume of pigment per unit projected area of skin were then obtained by multiplying these two parameters [39].

### Statistical analyses

To investigate whether animal colouration matches background colouration in free-living slugs in natural habitats, the mean R, G and B reflectance values from each slug were compared with those from the substrate upon which they were photographed. A one-way ANOVA was used to compare colour scores of slugs and substrate between sites of the same habitat type. Colour data of slugs and substrate were pooled for sites of the same habitat type (after it was determined that there were no significant differences between sites; Additional file 1: Table S1) and then tested for bivariate correlation using Pearson’s *r*. Students *t*-tests were used to test whether RGB reflectance values differed significantly between woodland and blanket bog substrate and between each of the two slug colour morphs. Students *t*-tests were also used to examine whether RGB reflectance scores, at the end of the experiment, differed between UV-treated slugs and slugs kept in darkness. Paired *t*-tests were used to test whether juvenile slugs differed in RGB reflectance scores after feeding trials were concluded. Data from stereological estimation of the % volume fraction of black pigment in epithelial sections were not normally distributed. A Mann-Whitney *U* test was therefore used to examine whether the % volume fraction of black pigment differed significantly between UV-irradiated and dark-reared slugs. A one-way ANOVA was used to test whether juvenile slugs differed in reflectance of R, G and B colour scores at the beginning of UV-exposure trials and feeding trials (to account for any potential variation between newly-hatched individuals). Graphs were prepared and statistical tests were carried out using SPSS (IBM, USA).

## Results

### Quantification of G. maculosus colour using digital photography

**Fig. 3:**
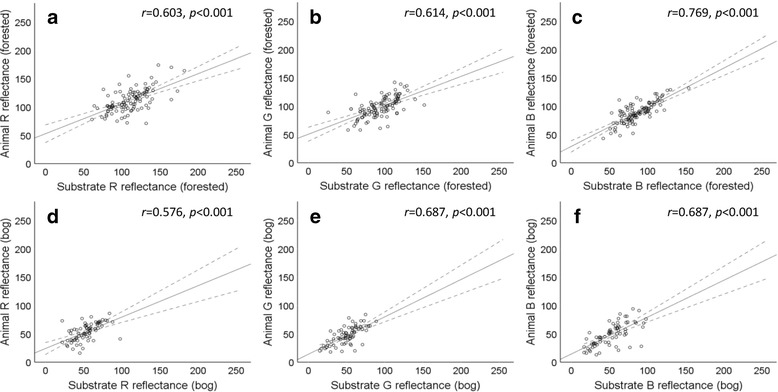
Correlations between animal and substrate R, G and B reflectance scores from forested (**a**–**c**; *n* = 104) and blanket bog (**d**–**f**; *n* = 71) habitats. Solid lines show Pearson’s *r* correlation; dotted lines show 95% CI

In total, 124 slugs were sampled from forested sites and 71 slugs were sampled from blanket bog sites. Colour scores did not differ significantly for slugs or substrate between sites of the same habitat type (with the exception of the B reflectance from forest slugs; Additional file 1: Table S2). Slugs from forest site 3 showed significantly lower mean B reflectance scores than slugs from both of the other forested sites. Given that subsequent analysis involved pooling of data, this site was removed from the data set – an explanation of why the B reflectance was significantly different in slugs from forest site 3 is given in the discussion. Colour scores of slugs and of substrates were pooled by habitat type (forest or bog). The R, G and B reflectance values of slugs and substrate were strongly and positively correlated for both forest and blanket bog habitats (Fig. 3). Mean R, G and B reflectance values of slugs differed significantly between habitats as did substrate (Table 1). R, G and B reflectance values did not differ significantly between slugs and substrate from the same habitat type but did differ significantly between slugs and substrate from the alternative habitat (i.e. between slugs from forest habitats and substrate from bog habitats, and between slugs from bog habitats and substrate from forest habitats; Table 2).

**Table 1:** Mean reflectance scores and *t* test statistics comparing RGB values of slug and substrate images from forested (*N* = 104 images) and blanket bog (*N* = 71 images) habitats

		Forested habitats		Blanket bog habitats		Between-habitats *t*-tests		
	Channel	Mean	SD	Mean	SD	Mean Diff.	*t*(173)	*p*
Slug	R	111.68	20.31	53.54	15.34	58.16	21.53	<0.001
	G	98.45	17.69	47.44	15.72	50.98	19.56	<0.001
	B	88.95	17.96	48.66	19.71	40.27	13.99	<0.001
Substrate	R	111.12	23.09	53.21	16.04	57.95	19.62	<0.001
	G	94.14	21.34	50.74	16.41	43.38	15.18	<0.001
	B	86.89	20.04	52.78	20.85	34.09	10.87	<0.001

**Table 2:** Results from *t* tests comparing mean RGB values of slugs against RGB values of substrate from their natural habitat, and against substrate from alternative habitats (sample sizes and means ± SD for each colour channel are presented in Table 1)

		Forested substrate			Bog substrate		
	Channel	Mean diff.	*t*	*p*	Mean diff.	*t*	*p*
Forested slug	R	0.56	0.18	0.852	−57.60	−19.80	<0.001
	G	4.30	1.58	0.115	−46.68	−16.64	<0.001
	B	2.05	0.78	0.437	−38.22	−12.46	<0.001
Bog slug	R	58.51	21.28	<0.001	0.35	0.13	0.894
	G	47.69	18.03	<0.001	−3.29	−1.22	0.224
	B	36.15	11.91	<0.001	−4.13	−1.21	0.227

### Effect of UV-exposure, darkness and diet on pigmentation

The R, G and B reflectance scores did not differ significantly between newly-hatched slugs before each experimental treatment (i.e. between UV and darkness treatments, and between different diet treatments; Additional file 1: Table S3).

**Fig. 4:**
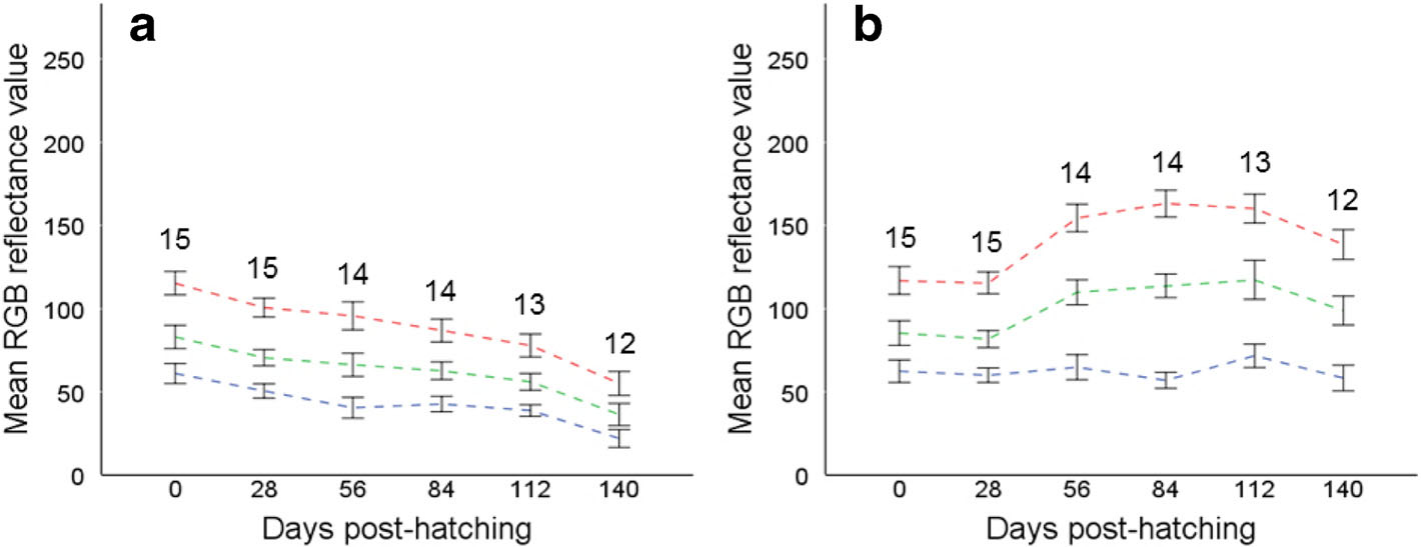
Juvenile slugs irradiated under UV-lighting (**a**) showed less reflectance in R (*top line*), G (*middle line*) and B (*bottom line*) colour channels with each month, becoming significantly darker than juveniles maintained in darkness (**b**). Colour reflectance scores differed significantly between UV-exposed and darkness reared slugs at the end of the experimental period (R: *t* = −14.461, *p* < 0.001; G: *t* = −11.391, *p* = <0.001; B: *t* = −7.700, *p* = <0.001). Numbers above means show group *n* at each sampling month; error bars show ±SE for means

**Fig. 5:**
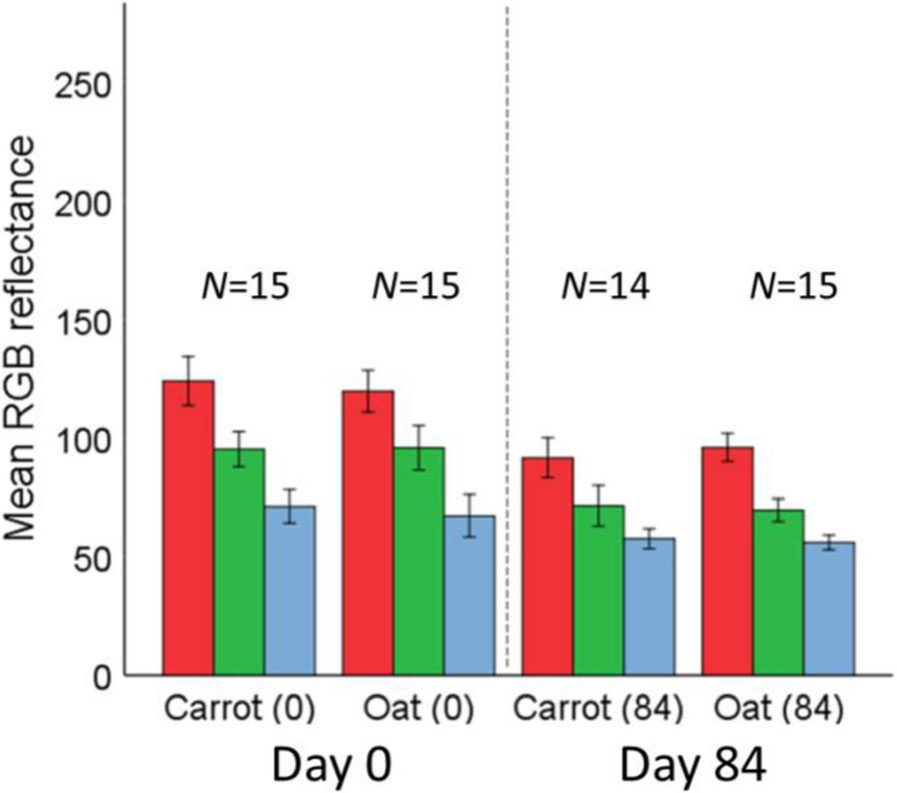
Juveniles reared on different diets showed significantly less reflectance in R, G and B colour channels (*shown as red, green and blue coloured bars*) after 84 days (*right of the dotted line*) than when they first hatched, but colour reflectance scores not differ significantly between diets after this period; (R: *t* = −0.876, *p* = 0.390; G: *t* = 0.409, *p* = 0.686; B: *t* = 0.612, *p* = 0.546). Error bars show ±2SE for group means; means ± SD given in Table 3

After a period of 140 days, UV-irradiated slugs displayed significantly lower R, G and B reflectance scores than when they first hatched (Fig. 4), whereas slugs reared in darkness did not differ significantly in colour reflectance scores after the same time period (Table 3). Slugs reared on different diets under laboratory conditions also exhibited significantly lower colour reflectance scores at the end of feeding trials (84 days) than when they first hatched (Table 3). However, colour reflectance scores did not differ significantly between diet treatments after a period of 84 days (comparable only for carrot and oat diets since juveniles reared on spinach died after 56 days; Fig. 5).

**Table 3:** Effect of different lighting and diet treatments on juvenile slug colouration from the time of hatching (start) to the end of the trials

			Start^a^		End^b^			
		Channel	Mean	SD	Mean	SD	*t*	*p*
		R	114.80	13.09	55.05	12.48	9.175	<0.001
	UV	G	83.26	11.31	36.26	11.65	7.879	<0.001
**Lighting**		B	60.64	10.44	21.86	9.40	7.455	<0.001
(fed on oats)								
		R	119.18	15.36	114.94	17.23	0.603	0.559
	Darkness	G	88.02	12.92	92.97	16.89	−1.179	0.263
		B	65.52	11.08	58.28	13.42	2.172	0.053
		R	124.07	16.35	91.68	13.21	6.27	<0.001
	Carrot	G	95.31	11.73	69.51	11.15	5.75	<0.001
		B	71.21	11.09	57.65	6.72	3.06	0.013
**Diet**		R	119.81	17.11	96.04	11.48	4.378	0.001
(natural photoperiod)	Oats	G	95.88	18.18	69.65	9.23	4.828	<0.001
		B	67.26	17.26	67.26	5.98	2.227	0.043

^a^Start = day of hatching, ^b^End = day 140 UV-exposure experiments, and day 84 for diet experiments

### Stereological estimation of epithelial pigment

**Fig. 6:**
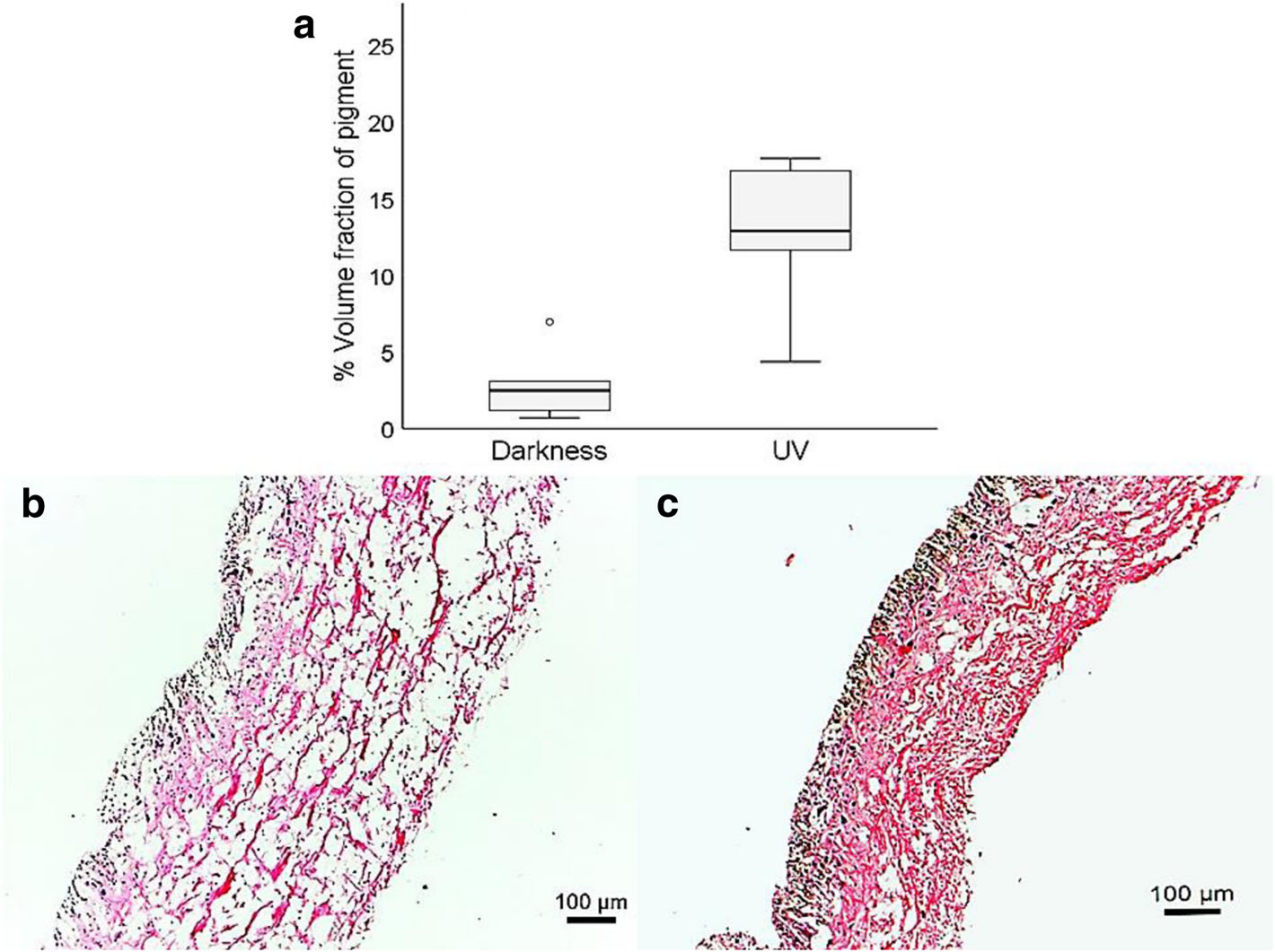
**a** UV-exposed slugs contained a significantly greater estimated volume of black pigment than darkness-reared slugs. Volume fraction estimates are expressed as percentages. TS of integument from **b** a darkness-reared (*brown*) juvenile and **c** a UV-exposed (*black*) juvenile (mag. ×200). Black pigment is concentrated in the outer epithelia of both slug colour morphs

Estimates of the volume fraction of black pigment (Fig. 6) were significantly greater in epithelial sections of UV-irradiated slugs (Mean rank: 9.33) than in darkness-reared slugs (Mean rank: 3.67; *U* (12) = 35, *p* = 0.004). There was no significant difference in mean epithelial thickness between slugs from each treatment group (*U* (12) = 30, *p* = 0.0649).

## Discussion

### Environment-phenotype matching

The colour values of adult slugs as estimated using digital photography are strongly and positively correlated with the colour properties of the substrate upon which they were found, in both forest and bog habitats. Furthermore, these colour values differed significantly between colour morphs and substrate from each habitat, and when animal colouration was compared to substrate colouration from the alternative habitat. This suggests a ‘mismatch’ between animal and substrate in alternative habitats and demonstrates that colouration in adult *G. maculosus* is habitat-specific. Brown ‘forest’ slugs may be mismatched on black and white ‘bog’ substrates and vice versa, suggesting that mismatched individuals would possess a lower degree of crypsis in the ‘wrong’ habitat type, possibly increasing their susceptibility to predation. A consistent pattern of background-phenotype matching is a good indication that a phenotype is adaptive [2]. The correlations found between R, G and B colour channels measured from *G. maculosus* and their substrate in both habitat types is consistent with the hypothesis that this species possesses camouflage which enables both colour morphs of *G. maculosus* to accurately match a random sample of their respective background. Hall et al. [40] demonstrated how disruptive markings can provide effective crypsis in multiple habitats. Spotted patterning, as well as background colour matching therefore likely provides strong, habitat-specific camouflage in *G. maculosus*.

Camouflage implies selective pressure from a visual predator. Although passerine birds are known to prey upon a number of medium-large arionid slug species [41], only one published record of predation exists for *G. maculosus* by the larval stage of the sciomyzid fly *Tetanocera elata* Fabricius, which appeared to initiate feeding upon reception of tactile or olfactory cues [42]. It is currently unknown whether the spotted patterns on both *G. maculosus* morphs act as disruptive markings against avian predators. Many birds are potentially tetrachromatic [43] and may therefore perceive colour in wavelengths undetected by this study (photography and image analysis methods used in this study provide a basic assessment of colouration in a trichromatic colour space - more accurate colour analysis would involve measuring differences in regions of a light spectrum, and mapping these to models representing the visual capacity of known predators [37]). A possible alternative explanation for spotted markings might be that they function in social signalling, as is known to be the case for other invertebrates (e.g. in wasps: [44, 45]). However, slugs are believed to have poor visual systems relative to many other invertebrate taxa and may be only capable of detecting the overall distribution of light and dark [46]. Furthermore, *G. maculosus* is hermaphroditic which makes a social signalling hypothesis for the spotted patterning unlikely. Allen [41] stated that visual predation by birds is undoubtedly the most important selective force in the evolution of cryptic colour morphs in prey animals and suggested that this explains why terrestrial gastropods in general living in forests tend to be brown. In addition to the pattern of background matching demonstrated by the results of this study, an unusual startle-response unique among gastropods to *G. maculosus* may also suggest selection by avian predators. Many slugs contract into a humped posture when disturbed [31]. However, *G. maculosus* curls up into a ball shape by contracting its foot completely in half, and secretes a low-viscosity mucus causing the animal to become more slippery (*pers. obs.*). This behaviour is perhaps most easily explained as an adaptation by increasing the difficulty of an avian predator to hold the prey in its bill.

### Colour change

Images of juvenile slugs used in feeding trials showed lower reflectance scores in all colour channels after 84 days. However, there was no statistically significant difference in R, G and B reflectance values between food treatments after the feeding trial period was concluded. Thus, even though juvenile slugs were darker after feeding trials, it seems to be independent of diet. Although the lichen composition found in the field differs between blanket bog and forested habitats, adult *G. maculosus* specimens from blanket bog habitats will eat similar foods to specimens from forested habitats (E. Johnston, *pers. comm.*) in captivity. While Jordaens et al. [27] showed how diet can influence body colour in slugs of the subgenus *Carinarion* Hesse, the slight darkening of juveniles observed after 84 days of feeding trials in this study is likely to be a natural result of growth. As in some other slug species (e.g. in *Limax flavus* [24]), newly hatched *G. maculosus* juveniles tend to be light brown in colour and somewhat translucent, so the darker colour values recorded after feeding trials were concluded are probably the result of increased overall size and thickening of the integument. Furthermore, the juvenile slugs used in feeding trials were maintained in plastic containers on a shelf in the laboratory approximately 5 m from a SW-facing window and, as such may have been exposed to a natural day/light cycle, which may also have influenced this slight darkening to some degree.

Slugs irradiated under UV-lighting became consistently darker at each sampling month and exhibited the black ‘bog’ morph after these trials were concluded, showing less reflectance in R, G and B colour channels. Slugs kept in darkness, however, remained lighter than UV-exposed juveniles at the end of the experimental period, showing higher reflectance scores in R, G and B colour channels. This result demonstrates that ultraviolet radiation can induce a change in pigmentation such that slugs exposed to UV radiation become darker in colour. Stereological examination of skin tissue from darkness-reared and UV-exposed juveniles demonstrated that UV-irradiated slugs contained significantly greater amounts of black pigment in their epithelium than darkness-reared slugs. This black pigment is most likely melanin, which has been detected in a number of other slug species (e.g. in *A. ater* [47]; *Arion hortensis* Férussac [48]; *Deroceras reticulatum* Müller [49]) and is known to develop in response to UV-exposure in a wide range of other animal taxa [50] but until this study, has not been demonstrated in slugs. Since juvenile *G. maculosus* from blanket bog habitats tend to be brown [31], it is likely that the black morph of adults develops in response to higher levels of UV exposure in these habitats, whereas juveniles which develop in relatively darker and more sheltered forested habitats remain brown. McCrone and Sokolove [51] showed that photoperiod was responsible for producing a maturation hormone in *Limax maximus* L., with long photoperiod exposures resulting in the development of male-phase, and shorter photoperiod exposures resulting in the development of female-phase reproductive morphologies upon maturation. A similar hormonal pathway may be present in *G. maculosus*, where black or brown pigmented phenotypes are expressed in response to different levels of UV radiation. Previously, adult *G. maculosus* specimens collected from open habitats appeared to lose some of their black pigment after a period of several weeks in the laboratory, with skin tissue in the grooves between tubercles becoming a paler brown colour (*pers*. *obs*.), suggesting that colour change may be reversible to some degree. However, it currently remains unclear whether colour change is completely reversible in adults or whether black pigmentation in *G. maculosus* is produced during a key period of early development in response to UV cues. UV radiation reaching the ground in bog habitats, particularly on overcast days and during sunrise/sunset hours when the slugs are most active, is likely to be lower than the UVB radiation emitted during laboratory experiments (25 μW/cm^2^). As such it remains unknown how long it takes juveniles to develop into the black morph in the field, and whether this change in pigmentation is completely or partially reversible. Slugs from forest site 3 were omitted from pooled analyses because they exhibited slightly lower mean reflectance scores in R and G channels, and significantly lower reflectance scores in B colour channels than slugs photographed from the other two forested sites. These slugs, although of the brown phenotype, appear to possess darker skin than slugs from the other forested sites. The area from which slugs were photographed from forest site 3 was noticeably brighter and had a luminosity value three times greater than the other two forest sites surveyed (Additional file 1: Table S1). This site also borders a blanket bog habitat, so it is possible that the slightly darker brown slugs photographed here were exposed to higher levels of UV light than slugs from the other two forested sites. This is consistent with a statement by Rowson et al. [31], that the distinction between brown and black *G. maculosus* colour forms becomes substantially blurred where wooded areas border open habitats.

### Origin of colour dimorphism

Reich et al. [32] demonstrated that *G. maculosus* originated during the middle Miocene, approx. 15Myr ago, probably arriving in Ireland from Iberia during the middle ages. The ability of *G. maculosus* to alter the degree of melanin-like pigment in its skin in response to UV exposure could have evolved relatively recently, during the Quarternary period. The Quarternary is characterised by the periodic growth and retreat of ice sheets across the northern hemisphere [52]. Hewitt [53] has suggested that animal and plant populations survived through several glacial cycles by migrating up and down mountains. Reich et al. [32] suggested that Iberian *G. maculosus* populations may have survived in mountain valleys during periods of glaciation when northern Spain’s mountain peaks would have been covered by ice. During warming phases when ice sheets were in retreat, *G. maculosus* populations would again have access to higher mountain altitudes, allowing them to increase their range altitudinally. Exposure to UV radiation can damage DNA and melanin can act as a protective filter in skin against the effects of UV exposure [54–56]. Migration by *G. maculosus* up and down mountain ranges over thousands of years as ice sheets periodically expanded and retreated may therefore have fixed in ancestral populations the ability to cope with different exposures to UV intensity. Dark populations of some slug species have previously been reported to prevail at high altitudes, possibly as a result of climatic selection (e.g. *A. ater* [21]; *A. rufus* and *A. lusitanicus* [26]; and *Lehmannia marginata* Müller [31]). However, pigmentation in these species is not known to be plastic. Since *G. maculosus* can self-fertilize, the capacity of an individual to express either black or brown phenotype in response to differential UV exposure may have been selected over non-plastic melanic morphs, which are known to occur as adaptations to altitudinal gradients in UV intensity in some lizards [12] and insects [57–59]. Skin colour in *G. maculosus* most likely plays a photoprotective role, leading to incidental camouflage against the different substrate types of each habitat. The spotted patterning present in both colour morphs likely affords *G. maculosus* with a degree of bet-hedging by increasing the effectiveness of this incidental camouflage in whichever habitat type it develops. Ahlgren et al. [60] found that the development of black skin pigmentation in the freshwater gastropod *Radix balthica* L. was induced by UV radiation and by the detection of kairomones from predatory fish, demonstrating how pigmentation may serve more than one function in cryptic animals. It is also possible that each *G. maculosus* colour morph has important implications for thermoregulation – with black slugs absorbing heat more efficiently than paler brown slugs, as has been shown for other ectothermic invertebrates on a wide geographic scale [61].

### Implications for other species

Dark colour morphs have been reported to prevail at high altitudes in many other terrestrial gastropod species, and this phenomenon has most often been explained as an example of climatic selection. It is possible that colouration is also a plastic trait in at least some of these species. Melanisation is correlated with a suite of behavioural traits in vertebrates: typically, darker vertebrates tend to be more aggressive, sexually active and resistant to stress than lighter vertebrates [62]. Although melanin development pathways differ significantly between vertebrates and invertebrates [63], further research into the links between pigmentation and behaviour may reveal many analogues from invertebrate systems – particularly in species with melanin-based colour polyphenisms. Results from behavioural studies with *G. maculosus* have shown that black slugs collected from bog habitats exhibit a significantly faster escape response (data to be published elsewhere), as well as a greater degree of sinuosity in food-searching behaviour than brown individuals collected from forests (E. Johnston, *pers*. *comm*.), demonstrating greater levels of boldness and exploratory behaviour, respectively. Such consistent intraspecific behavioural types may also be common to other slugs, and studying their occurrence could have useful practical implications. For example, the grey field slug *D. reticulatum* is a major agricultural pest which can be difficult to identify due to its highly variable skin colour - it occurs on a spectrum of very pale to deep brown-coloured individuals, even within populations [31]. Luther [22] demonstrated that pigmentation in *D. reticulatum* is genetically controlled, with melanised forms dominant to unpigmented individuals. However, the degree of melanisation may well be influenced by UV exposure in darkly pigmented *D. reticulatum*, as has been presently demonstrated for *G. maculosus*. Furthermore, Chevallier [26] reported that populations of the highly invasive slug *A. lusitanicus* are darker at high altitudes, citing it as a case of climatic selection. It is likely that darker forms are at least in part melanised due to higher UV-exposure for *A. lusitanicus*, *D. reticulatum* and a host of other terrestrial slugs in which colour polyphenisms were previously believed to be the sole result of climatic selection or putatively non-adaptive genetic inheritance. It may therefore be useful for researchers interested in developing pest control protocols to investigate whether melanisation could also be used as a predictor of boldness or exploratory behaviour in pestiferous and invasive slugs.

## Conclusions

Colour dimorphism in *G. maculosus* is consistent with the idea of habitat-specific crypsis, with a likely additional function for photoprotection. Adult pigmentation is a plastic trait determined by differential exposure to UV radiation and colour dimorphism in this species may have initially originated as an adaptation to clinal differences in UV radiation. Phenotypic plasticity in *G. maculosus* pigmentation could represent a generalist strategy to reduce detectability by visual predators by providing incidental crypsis in different habitat types and experiments are planned to test this hypothesis with passerine birds. To our knowledge, the results from this study provide the first evidence of plastic colour change in a terrestrial mollusc in response to UV radiation. Recently, *G. maculosus* populations been recorded from a number of plantation forests throughout its Irish distribution [33–35, 64], and forest clear-felling may significantly reduce population sizes in these habitats. The ability of *G. maculosus* to change colour with UV-exposure may also have important implications for the conservation and management of this protected species. Slugs which remain in clear-felled areas should develop the black ‘bog’ phenotype upon exposure to the relatively higher levels of UV post-felling. We would expect these black phenotypes to provide ineffective camouflage against a tree-stump background, which may reduce their fitness by making the slugs more conspicuous to visually-foraging predators. Careful consideration therefore needs to be given to site and habitat selection where translocation of *G. maculosus* populations may be used as a mitigation measure for forestry activities.

## Additional files

Additional file 1: **Table S1**. Description of study sites. **Table S2**. Results of a one-way ANOVA comparing mean slug and substrate RGB reflectance values between sites of the same habitat type. **Table S3**. Results of a one-way ANOVA comparing mean RGB reflectance values between groups prior to diet experiments; and results of an independent samples *t* test comparing RGB reflectance values between groups prior to UV and darkness experiments. (DOCX 18 kb)

Additional file 2: Epithelial stereology. (XLSX 28 kb)

Additional file 3: Feeding trials. (XLSX 14 kb)

Additional file 4: Lighting trials. (XLSX 18 kb)

Additional file 5: Slug and Substrate RGB reflectance values. (XLSX 21 kb)
