# Supplementary material for: Quantifying phenotype-environment matching in the protected Kerry spotted slug (Mollusca: Gastropoda) using digital photography: exposure to UV radiation determines cryptic colour morphs
Source: Front Zool. 2017 Jul 10;14:35. doi: 10.1186/s12983-017-0218-9 (PMC5504635; doi:10.1186/s12983-017-0218-9)
Supplement: Supplementary file 1 — Table S1. Description of study sites. Table S2. Results of a one-way ANOVA comparing mean slug and substrate RGB reflectance values between sites of the same habitat type. Table S3. Results of a one-way ANOVA comparing mean RGB reflectance values between groups prior to diet experiments; and results of an independent samples t test comparing RGB reflectance values between groups prior to UV and darkness experiments. (DOCX 18 kb) [file 12983_2017_218_MOESM1_ESM.docx]

**Supplementary Information**

Table S1. Description of study sites

| Site name | Site no. | Coordinates | Altitude | Habitat type | KLx | Dominant vegetation |
| --- | --- | --- | --- | --- | --- | --- |
| Oughterard | 1 | N53˚22ʹ33.2ʺ W9˚24ʹ40.9ʺ | 195m | Conifer forest | 0.98 | *Picea sitchensis* Carr. |
| Oughterard | 2 | N53˚21ʹ49.5ʺ  W 9˚25ʹ17.3ʺ | 199m | Blanket bog | 14.46 | *Molinia caerulea* L & granite boulder outcrops |
| Tooreenafersha | 3 | N51˚54ʹ7.1ʺ W9˚47ʹ18.9ʺ | 110m | Conifer forest | 2.91 | *Picea sitchensis* Carr. |
| Uragh | 4 | N51˚48ʹ5.7ʺ W9˚40ʹ34ʺ | 50m | Blanket bog | 11.68 | *Molinia caerulea* L & sandstone boulder outcrops |
| Glengarriff | 5 | N51˚45ʹ14.8ʺ W9˚34ʹ2.2ʺ | 19m | Deciduous woodland | 0.88 | *Quercus patraea* Liebl |
| Leahill | 6 | N51˚42ʹ1.6ʺ W9˚37ʹ17.2ʺ | 77m | Blanket bog | 15.01 | *Molinia caerulea* L & sandstone boulder outcrops |

Altitude=m above sea level, KLx=luminosity measured from centre of each site on first day of sampling

Table S2. Results of a one-way ANOVA comparing mean slug and substrate RGB reflectance values between sites of the same habitat type

|  | | | ^a^Forested sites (*N*=3) | | ^b^Blanket bog sites (*N*=3) | | |
| --- | --- | --- | --- | --- | --- | --- | --- |
|  | Channel | F(2, 121) | | *p* |  | F(2, 68) | *p* |
|  | R | 1.877 | | 0.157 |  | 0.330 | 0.568 |
| Slug | G | 2.349 | | 0.100 |  | 0.671 | 0.416 |
|  | B | 4.003 | | 0.021 |  | 2.293 | 0.135 |
|  |  |  | |  |  |  |  |
|  | R | 1.507 | | 0.226 |  | 2.389 | 0.127 |
| Substrate | G | 2.593 | | 0.079 |  | 0.206 | 0.652 |
|  | B | 2.394 | | 0.096 |  | 0.010 | 0.921 |

^a^*N*=124 images of slug/substrate from forested sites; ^b^*N*=71 images of slug/substrate from blanket bog sites

Table S3. Results of a one-way ANOVA comparing mean RGB reflectance values between groups prior to diet experiments; and results of an independent samples *t* test comparing RGB reflectance values between groups prior to UV and darkness experiments

|  | | | Diet groups (*N*=3) | | UV+darkness (*N*=2) | | |
| --- | --- | --- | --- | --- | --- | --- | --- |
|  | Channel | F(2) | | *p* |  | *t* | *p* |
|  | R | 0.308 | | 0.737 |  | -0.297 | 0.769 |
| Slug | G | 0.129 | | 0.879 |  | -0.461 | 0.648 |
|  | B | 0.214 | | 0.809 |  | -0.305 | 0.763 |
| Means ± SD are presented in text in Table 3 | | | | | | | |
